# Prohibitive Effects of ω‐3 on Pancreatic Damage in an Experimental Diabetes Model: Blood Glucose, Insulin, Lipid Profiles, Oxidative Stress, Inflammation, Pancreatic Tissue Morphology, and Apoptotic Pathways

**DOI:** 10.1002/jbt.71154

**Published:** 2026-10-25

**Authors:** Zeynep Erdemli, Mehmet Gul, Nurcan Gokturk, Fatma Ozhilal, Edanur Ozsoy, Harika Gozukara Bag, Mehmet Erman Erdemli

**Affiliations:** ^1^ Department of Medical Biochemistry Inonu University Malatya Türkiye; ^2^ Department of Histology and Embryology Inonu University Malatya Türkiye; ^3^ Department of Histology and Embryology Malatya Turgut Ozal University Malatya Türkiye; ^4^ Department of Biostatistics Inonu University Malatya Türkiye

**Keywords:** apoptosis, diabetes, inflammation, omega‐3, oxidative stress, pancreas

## Abstract

This study aimed to investigate the potential protective effects of Omega‐3 (ω−3) against damage to pancreatic tissue in rats with experimentally induced diabetes using streptozotocin. Twenty‐eight male Wistar Albino rats were divided into four groups: Control, Diabetes Mellitus (DM), ω−3, and DM + ω−3, with seven rats in each group. STZ was injected intraperitoneally (i.p.) into the DM group, and blood glucose levels were measured on the fourth day after injection. Rats with blood glucose levels of ≥ 200 mg/dL were diagnosed with DM. The ω−3 group received 500 mg/kg of ω−3. After the 42‐day experiment, pancreatic tissue and blood samples were collected from the rats. The DM group showed increased levels of malondialdehyde (MDA), total oxidant status (TOS), oxidative stress index (OSI), tumor necrosis factor α (TNF‐α), interleukin‐6 (IL‐6), glucose, LDL, triglycerides, caspase‐3 immunoreactivity, and histopathological abnormalities in pancreatic tissue. At the same time, there were decreases in superoxide dismutase (SOD), catalase (CAT), total antioxidant status (TAS), HDL, and insulin levels. Blood, biochemical, histopathological, and immunohistochemical parameters negatively impacted by ω−3 administration have improved. This study demonstrated that in STZ‐induced DM rats, DM increased oxidative capacity and led to oxidative stress, inflammation, apoptosis, and tissue damage. It was found that ω−3 has strong antioxidant, pro‐apoptotic, anti‐inflammatory, and protective effects.

## Graphical abstract

These omega 3 and duration doses have been studied for the first time in the literature. At this dose and duration, omega 3 has shown very strong antioxidant, anti‐inflammatory and apoptotic effects. Omega‐3 can be used to alleviate diabetes and diabetes‐related complications.

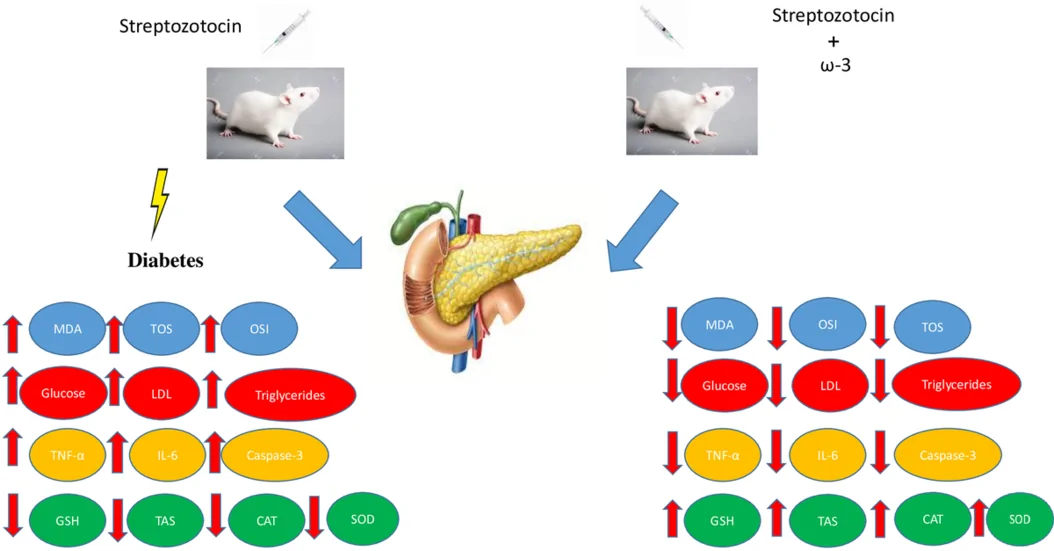

## 1 Introduction

Diabetes mellitus (DM) is one of the most common health problems in modern society. The number of people with diabetes keeps increasing every day. According to 2017 data from the International Diabetes Federation (IDF), there are 451 million people worldwide with diabetes, which is about 1 in every 11 people, and many remain undiagnosed. Although it is estimated that this number will reach 693 million by 2045, it could be much higher [1]. 10% of global health expenditures—US$760 billion—are spent on diabetes. DM is a chronic endocrine disorder characterized by high blood sugar levels. Hyperglycemia disrupts the metabolism of carbs, proteins, and fats, leading to various harmful processes. These processes cause autoimmune destruction of pancreatic β‐cells, resulting in insulin deficiency. When insulin doesn't work properly on target tissues, it causes damage and dysfunction in many parts of the body, including the eyes, kidneys, nerves, heart, blood vessels, and urogenital system. Long‐term complications of diabetes include retinopathy, which can cause vision loss; nephropathy, which can lead to kidney failure; peripheral neuropathy; and autonomic neuropathy, which can cause gastrointestinal, genitourinary, cardiovascular symptoms, sexual dysfunction, and increased oxidative stress [2, 3]. Streptozotocin (STZ) is a compound naturally produced by the soil bacterium *Streptomyces achromogenes*. Its ability to induce diabetes was discovered in 1963, and it has since been used in experimental animal studies to produce diabetes [4, 5, 6, 7, 8].

Interest in omega‐3 (ω−3) polyunsaturated fatty acids (PUFAs) has grown recently because of their various roles in promoting health and lowering disease risk. ω−3 fatty acid types can have different effects at the molecular level [9, 10] on various mechanisms. Specifically, ALA, EPA, and DHA have been shown to regulate glucose balance and insulin sensitivity [11, 12, 13]. ω−3 PUFAs are important for adjusting blood lipid profiles and membrane lipid composition, as well as influencing eicosanoid biosynthesis, cell signaling pathways, and gene expression. Additionally, ω−3 PUFAs provide benefits for cardiovascular disease, diabetes, cancer, and depression [14, 15, 16].

The current study investigated the potential protective and reparative effects of ω−3 fatty acids on pancreatic tissue damage in rats with experimental streptozotocin (STZ)‐induced diabetes.

## 2 Materials and Methods

### 2.1 Experimental Animals

Twenty‐eight 12‐week‐old male Wistar Albino rats, weighing between 200 and 300 g, were divided into four groups. All authors confirm that all animal experiments comply with the ARRIVE guidelines and are conducted in accordance with the UK Animals (Scientific Procedures) Act of 1986 and related guidelines, EU Directive 2010/63/EU for animal experiments, or the National Institutes of Health guide for the care and use of laboratory animals (NIH Publications No. 8023, revised 1978). The rats were housed on a 12‐h light‐dark cycle in air‐conditioned rooms maintained at 22°C with 55%–60% humidity. They had unrestricted access to food and water. The rats were fed a pellet diet free of phytoestrogens, containing 17% crude protein, 5.5% fat, 4.8% crude fiber, 7.2% crude ash, with at least 1.07% lysine, 0.64% methionine, at least 0.36% cysteine, a minimum of 25,000 IU/kg of vitamin A, and at least 70 mg/kg of vitamin E. Water bottles were made from glass, and the cages for the rats were made from BPA‐free plastics. Food and water were provided ad libitum.

### 2.2 Experimental Design

Rats were randomly assigned to Control (C), DM, ω−3, and DM+ ω−3 groups, with 7 rats in each group. DM was induced by a single injection of 50 mg/kg STZ dissolved in saline (SF), and rats were considered diabetic if their blood glucose levels increased at least twofold by the 4th day after injection.

**Control Group**: This group received standard feed and water available ad libitum.

**DM Group:** STZ (50 mg/kg), dissolved in SF, was administered as a single intraperitoneal dose, and the rats had free access to food for 42 days.

**ω−3 Group:** This group received 500 mg/kg of ω−3 orally each day for 42 days.

**DM** + **ω−3 Group:** Diabetes was induced by a single intraperitoneal injection of STZ (50 mg/kg) dissolved in SF, and 500 mg/kg of ω−3 was administered orally for 42 days.

For STZ, CAYMAN's Streptozotocin 13104‐1 was used, and for ω−3, EFA Liquid (280 mg/ml ω−3; 85 mg/ml EPA; 148 mg/ml DHA) was used.

### 2.3 Collection of Tissue Samples

At the end of the 42‐day experimental period, the rats' body weight and blood glucose levels were measured, and they were administered 5 mg/kg of xylazine and 50 mg/kg of ketamine intraperitoneally. Under anesthesia, the rats underwent a midline abdominal incision, and blood samples were collected from the posterior vena cava. After centrifuging the collected blood samples at 500 g for 5 min, the separated serum was transferred to Eppendorf tubes and stored at −77°C for biochemical analysis. Excess blood in the pancreatic tissue was removed with saline solution. The samples were stored in a deep freezer at −77°C for biochemical measurements and placed in 10% formalin for histopathological procedures.

### 2.4 Biochemical Analysis

Pancreatic tissues were removed from the freezer and weighed immediately before thawing. Nine times their weight in phosphate buffer was added to the tissues. Homogenization was performed at +4°C for 1 min at 16,000 rpm (IKA, Germany). The levels of malondialdehyde (MDA) in these homogenates were measured. The homogenates were centrifuged at 4°C and 500 g for 20 min to obtain the supernatants. From these supernatants, the following were measured: glutathione (GSH), superoxide dismutase (SOD), catalase (CAT), total oxidant capacity (TOS), total antioxidant capacity (TAS), oxidative stress index (OSI), and protein levels.

### 2.5 MDA

1.5 mL of 1% H_3_PO_4_ and 0.5 mL of 0.6% thiobarbituric acid (TBA) were mixed with 0.25 mL of tissue homogenate. The mixture was heated to 30°C to ensure complete dissolution of TBA, then heated at 100°C for 40 min. After 40 min, a red color appeared in the tubes. After cooling the tubes in tap water, 2 mL of n‐butanol was added to each sample. Each tube was vortexed for 5 min, then the samples were centrifuged at 25°C at 4500 rpm for 30 min. The supernatant (250 μL), which was the n‐butanol phase, was carefully removed and placed into separate quartz microplate wells. Readings were taken at 535 nm. The results were expressed as nmol/gram wet tissue [17].

### 2.6 Reduced GSH

The supernatant was deproteinized with tricarboxylic acid (TCA). For this, 125 µL of TCA was added to 125 µL of the sample. After vortexing thoroughly for 25 min, the mixture was centrifuged at +4°C and 4500 rpm to obtain a protein‐free supernatant. Each sample was then added to separate microplate wells in this order: 29 µL of deproteinized sample, 29 µL of DTNB, and 235 µL of Na_2_HPO_4_. After a brief vortex, it rested for 5 min. Measurements were taken at 410 nm within 5 min. The results are expressed as nmol/gram wet tissue [18].

### 2.7 SOD

9.13 mg of xanthine was dissolved in 200 mL of distilled water. Additionally, 23 mg of Na_2_EDTA was dissolved in 100 mL of distilled water; 12.3 mg of nitro blue tetrazolium (NBT) was dissolved in 100 mL of distilled water; 30 mg of bovine serum albumin (BSA) was dissolved in 30 mL of distilled water; and 2.54 g of Na_2_CO_3_ was dissolved in 60 mL of distilled water to prepare the stock solution. A mixture of 2.35 mL of this stock solution, 50 µL of xanthine oxidase (XO), and 500 µL of the sample was placed into propylene tubes and thoroughly vortexed. The tubes were incubated for 25 min, after which 1 mL of CuCl_2_ was added. SOD activity was determined by measuring the absorbance of the blue formazone at 560 nm. Distilled water served as the blank. Results are expressed as U/g protein [19].

### 2.8 CAT

A total of 4.205 g of potassium dihydrogen phosphate (KH_2_PO_4_) and 2.71 g of disodium hydrogen phosphate (Na_2_HPO_4_) were dissolved in 1 L of distilled water. Hydrogen peroxide (H_2_O_2_) was then added to this buffer, and the UV spectrum was adjusted at 240 nm. The decrease in absorbance was recorded over 1 min for 10 µL of sample in 2990 mL of phosphate buffer containing H_2_O_2_ using kinetic readings to measure enzyme activity. The enzyme activity is expressed as K/g protein [20].

### 2.9 Protein

Protein content in tissues is essential for measuring enzyme activities. The Lowry method was used to determine tissue protein levels. The absorbance of the resulting color was measured with a 660 nm spectrophotometer to assess protein content [21].

### 2.10 TAS

TAS was measured using the TAS Rel Assay kit (Rel Assay Diagnostics, Gaziantep, Turkey). The measurement was based on the principle of decolorizing antioxidant molecules. The steps were followed sequentially according to the kit instructions. First, the instrument was set to 37°C, shaken for 7 s, allowed to pause for 23 s, and read at 660 nm. 12 μL of samples were added to the wells. Level 1, Level 2, and standards were added to separate wells, three wells each. Then, 200 μL of reagent 1 was added to these wells, and the first reading was taken immediately at 660 nm. Next, the instrument was set to 37°C, shaken for 10 s, allowed to pause for 4.30 min, and read again at 660 nm. 30 μL of reagent 2 was added to each well, and the reading was taken again. Calculations were performed as specified in the kit instructions. Results are expressed as mmol Trolox equivalents per liter [22].

### 2.11 TOS

TOS was measured using the TAS Rel Assay kit (Rel Assay Diagnostics, Gaziantep, Turkey). Ferric ions form a colored complex with a chromogenic solution by converting ferrous ion chelator complexes of oxidants in the sample into ferric ions. As indicated in the kit, the TOS was determined by measuring the absorbance of the colored complex at 530 nm with the instrument set to 25°C. Results are expressed as μmol H_2_O_2_ equiv/L [23].

### 2.12 Serum

The levels of serum biochemical parameters (glucose, insulin, triglycerides, total cholesterol, LDL, and HDL) were measured using commercial Architect c1600 automatic analyzer kits from Abbott (Abbott Park, IL, USA).

### 2.13 Inflammatory Analysis

TNF‐α and IL‐6 levels were determined using rat ELISA kits (Immuno‐Biological Laboratories Co. Ltd., Takasaki Shi, Japan) according to the manufacturer instructions. The results are presented as ng/L.

### 2.14 Histopathological and Immunohistochemical Analysis

Four µm‐thick sections were stained using immunohistochemistry (IHC) with the primary antibody caspase‐3 (3C119): sc‐70497 (Santa Cruz Biotechnology Inc.). Four‐micrometer‐thick sections of pancreas were placed on slides and deparaffinized. The slides were then placed in a citrate buffer solution (PBS, Santa Cruz Biotechnology Inc., sc‐294091) and subjected to antigen retrieval in a pressure cooker (Retriever 2100, Prestige Medical Ltd., Blackburn, Lancashire, UK) at 120°C for 15 min. After cooling, the citrate buffer was removed and replaced with PBS. The slides were washed with PBS at each step, except between protein blocking and primer antibody application. A horseradish peroxidase detection kit (Santa Cruz Biotechnology Inc.) for immunohistochemical staining was used according to the manufacturer's protocol. First, 3% hydrogen peroxide was applied for 10 min. Next, the slides were incubated in a protein V blocking solution for 5 min, followed by a 1‐h incubation with an anti‐caspase‐3 primary antibody (Santa Cruz Biotechnology Inc., dilution 1:300). Third, the slides were incubated in a biotinylated goat anti‐polyvalent secondary antibody for 10 min. Then, streptavidin peroxidase was applied and incubated for 10 min. The slides were subsequently incubated with AEC chromogen (Santa Cruz Biotechnology Inc.) for 15 min. Finally, the slides were immersed in distilled water, counterstained with Mayer's hematoxylin, and mounted. All sections examined by the blinded histologist were viewed using a Nikon Eclipse Ni‐U light microscope with a DS‐Fi3 camera attachment and analyzed with the Nikon NIS‐Elements Documentation 5.02 image analysis system (Nikon Corporation, Tokyo, Japan).

Histopathological damage was scored in the examined pancreatic sections, including endocrine pancreatic cells (hydropic vacuoles in the cytoplasm, heterochromatic‐picnotic nuclei, and endocrine cell degeneration) and exocrine pancreatic tissue (cytoplasmic vacuolization, damage, degeneration, and necrosis in acinar cells). The Schmidt standards were modified as follows: Acinar cell damage in the exocrine pancreas: 0, absent; 1, focal (> 1% and < 10%); 2, sub‐lobular (> 10% and < 20%); 3, lobular (> 20% and < 40%); 4, lobular (> 40% and < 80%); 5, lobular (> 80%). The cellular damage scored in endocrine pancreatic cells included hydropic vacuoles in the cytoplasm, heterochromatic‐picnotic nuclei, cell degeneration, and necrosis: 0, absent; 1, involving > 1% and < 10% of islet cells; 2, > 10% and < 20%; 3, > 20% and < 40%; 4, > 40% and < 80%; 5, > 80%.

The H‐score method was used to assess the level of caspase‐3 immunoreactivity in both the endocrine and exocrine tissues of the pancreas. This semi‐quantitative immunohistochemical technique involves multiplying the percentage of positively stained cells by a weighted measure of staining intensity: H score = Ʃ Pi (i + 1), where Pi represents the percentage of stained cells at each intensity level (0%–100%). The staining intensity was characterized as absent; 0, weak but detectable above control; 1, clear; 2, and very strong; 3 [24].

### 2.15 Statistical Analysis

The normality of the quantitative data was evaluated using the Shapiro‐Wilk test. Variables that were normally distributed are presented as mean ± standard deviation, while others are shown as median (interquartile range). Homogeneity of variances within groups was tested with Levene's test. Normally distributed variables with equal variances were compared using one‐way analysis of variance and Tukey's post hoc test. Those with unequal variances were compared with Welch's test and Tamhane's T2 post hoc test. Variables that were not normally distributed were analyzed with the Kruskal‐Wallis test and Conover's post hoc test. The results of pairwise comparisons are indicated by superscript letters in APA style; differences between groups with different superscripts are statistically significant. A two‐sided significance level of 0.05 was used in all analyses.

## 3 Results

### 3.1 Biochemical and Inflammation

The MDA, TOS, and OSI levels were higher in pancreatic tissues from the diabetes group than in those from the other groups (Table 1). Blood samples also showed increased levels of glucose, LDL, triglycerides, total cholesterol, TNF‐α, and IL‐6. In the diabetes group, compared to all other groups, significant decreases were observed in GSH, SOD, CAT, and TAS levels in pancreatic tissues, as well as insulin levels in blood samples. In the ω−3 group, significant increases were observed in GSH, SOD, CAT, and TAS in pancreatic tissue compared to all other groups. There were decreases in TNF‐α and IL‐6 levels, along with reductions in glucose, triglycerides, LDL, and total cholesterol levels in the blood samples. HDL levels in blood samples increased. Insulin levels remained similar to those of the control group. In the diabetes group, coadministration of ω−3 resulted in decreases in MDA and TOS levels, along with reductions in TNF‐α, IL‐6, LDL, and triglyceride levels, while increases were observed in SOD, CAT, GSH, TAS, and HDL levels compared to the DM group (Tables 2, 3, 4).

**Table 1:** Oxidant‐antioxidant parameters.

	MDA (nmol/gwt)	GSH (nmol/gwt)	SOD (U/g protein)	CAT (K/g protein)
Control	261.96^a^ ± 23.72	940.25^a^ ± 47.66	3.30^a^ ± 0.05	1.53^a^ ± 0.05
ω−3	216.28^b^ ± 42.32	1242.66^b^ ± 28.59	5.44^b^ ± 0.07	1.84^b^ ± 0.13
DM	386.32^c^ ± 36.04	653.29^c^ ± 22.48	2.24^c^ ± 0.11	0.88^c^ ± 0.22
DM + ω‐3	294.64 ^d^ ± 34.56	1025.3 ^d^ ± 50.53	3.78 ^d^ ± 0.23	1.25^ac^ ± 0.24
*p*‐value	< 0.001	< 0.001	< 0.001	< 0.001

*Note:* Data are expressed as mean ± standard deviation. There is a statistically significant difference between groups marked with different superscript letters. Abbreviations: CAT, catalase; GSH, reduced glutathione; gwt, gram wet tissue; MDA, malondialdehyde; SOD, superoxide dismutase.

**Table 2:** Inflammation parameters.

	TNF‐α (ng/L)	IL‐6 (n/gL)
Control	7.67^a^ ± 0.17	5.11^a^ ± 0.14
ω−3	6.93^b^ ± 0.32	5.06^a^ ± 0.17
DM	18.7^c^ ± 0.67	11.94^b^ ± 0.24
DM + ω−3	9.64 ^d^ ± 0.79	7.1^c^ ± 0.31
*p*‐value	< 0.001	< 0.001

*Note:* Data are expressed as mean ± standard deviation. There is a statistically significant difference between groups marked with different superscript letters.

Abbreviations: IL‐6, interleukin‐6; TNF‐α, tumor necrosis factor‐alpha.

**Table 3:** Oxidative stress index parameters.

	TOS (μmol/L)	TAS (mmol/L)	OSI (AU)
Control	11.25^a^ ± 0.39	1.56^a^ ± (0.09)	71.27^a^ ± 3.73
ω−3	6.34^b^ ± 0.25	4.56^b^ ± (0.32)	13.86^b^ ± 1.09
DM	28.12^c^ ± 3.35	0.93^c^ ± (0.3)	268.42^c^ ± 47.55
DM + ω−3	14.12 ^d^ ± 1.92	2.13 ^d^ ± (0.36)	62.74^a^ ± 15.47
*p*‐value	< 0.001	< 0.001	< 0.001

*Note:* Data are expressed as mean ± standard deviation. There is a statistically significant difference between groups marked with different superscript letters.

Abbreviations: OSI, oxidative stress index; TAS, total antioxidant status; TOS, total oxidant status.

**Table 4:** Serum glucose and lipid profiles parameters.

	Glucose (mg/dL)	Insulin (µU/mL)	Triglyceride (mg/dL)	Total cholesterol (mg/dl)	LDL (mg/dL)	HDL (mg/dL)
Control	100.71^a^ ± 1.25	20.29^a^ ± 1.60	50^a^ ± 1	62.57^a^ ± 1.72	11^a^ ± 1	42^a^ ± 3
ω−3	96.29^b^ ± 2.63	20.0^a^ ± 1.16	42^b^ ± 3	59.57^a^ ± 3.82	6^b^ ± 1	57^b^ ± 2
DM	250.0^c^ ± 3.37	4.14^b^ ± 1.35	77^c^ ± 6	87.29^b^ ± 1.38	17^c^ ± 1	33^c^ ± 8
DM + ω−3	116.43 ^d^ ± 5.13	6.0^b^ ± 1.29	53 ^d^ ± 4	65.00^a^ ± 1.16	13 ^d^ ± 1	39^c^ ± 2
*p*‐value	< 0.001	< 0.001	< 0.001	< 0.001	< 0.001	< 0.001

*Note:* Data are expressed as mean ± standard deviation. There is a statistically significant difference between groups marked with different superscript letters.

### 3.2 Histopathological and Immunohistochemical Findings

Light microscopy showed that most of the pancreatic tissue was made up of acinar and ductal structures. Endocrine areas were present within the exocrine tissue as clusters of endocrine cells arranged in islet‐like formations. In the pancreatic sections of rats in the control group (Figure 1a–c) and the omega‐3 group (Figure 2a–c), the exocrine pancreatic acini, duct units, and endocrine islets appeared to have normal histological structures. Under light microscopy, most pancreatic sections consisted of exocrine tissue, while endocrine sections appeared as isolated cell clusters within the pancreatic lobules, forming islets. H&E‐stained pancreatic sections from rats in the control (Figure 1a–c) and Omega‐3 groups (Figure 2a–c) showed normal histological features.

**Figure 1:**
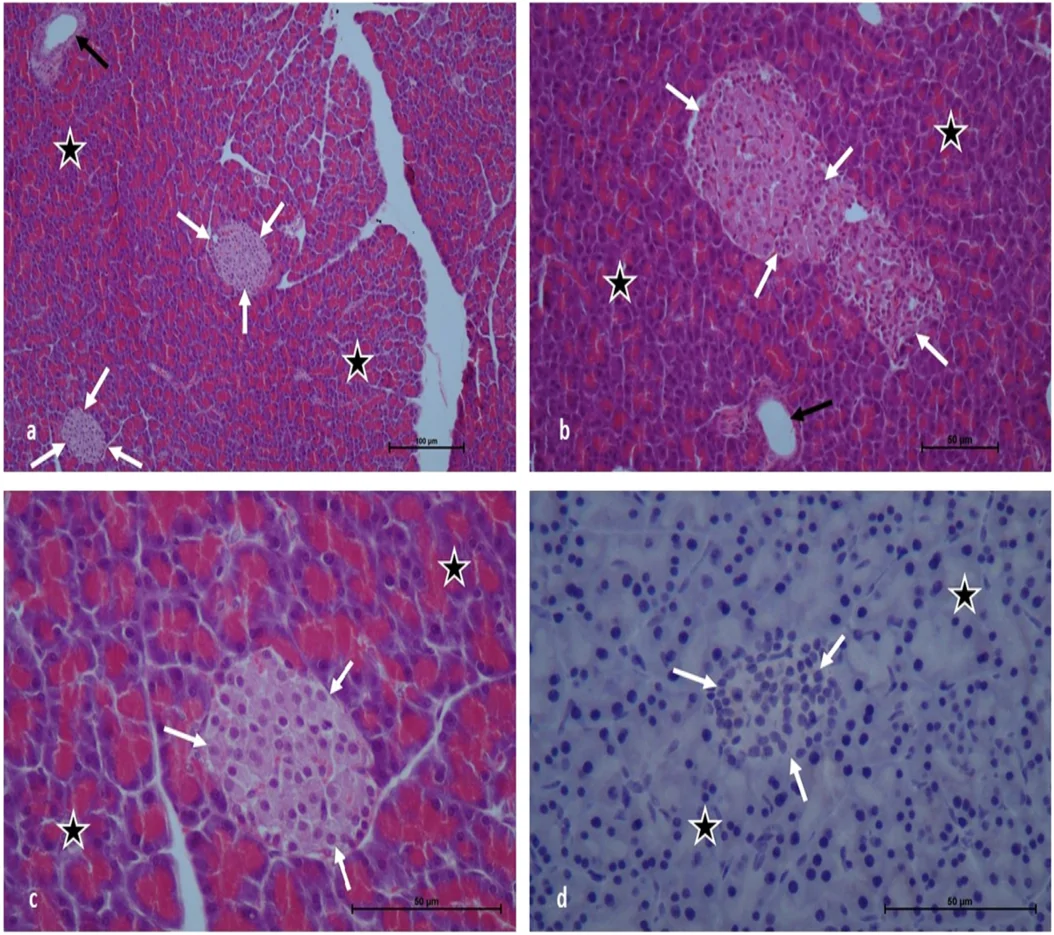
Control group Histopathological images (a) Control group: Normal histological appearance of the endocrine (white arrows) and exocrine (aster) pancreas, pancreatic duct (black arrow). H&E, X10. (b) Control group: Endocrine pancreas (white arrows), exocrine pancreas (aster) and pancreatic duct (black arrow) with normal histological appearance. H&E, X20. (c) Control group: Endocrine pancreas islet (arrows) and exocrine pancreas acini (aster) with normal histological appearance. H&E, X40. (d) Control group: Negative caspase‐3 immunoreactivity in endocrine pancreatic cells (arrows), exocrine pancreatic acini (aster). IHC, X40.

**Figure 2:**
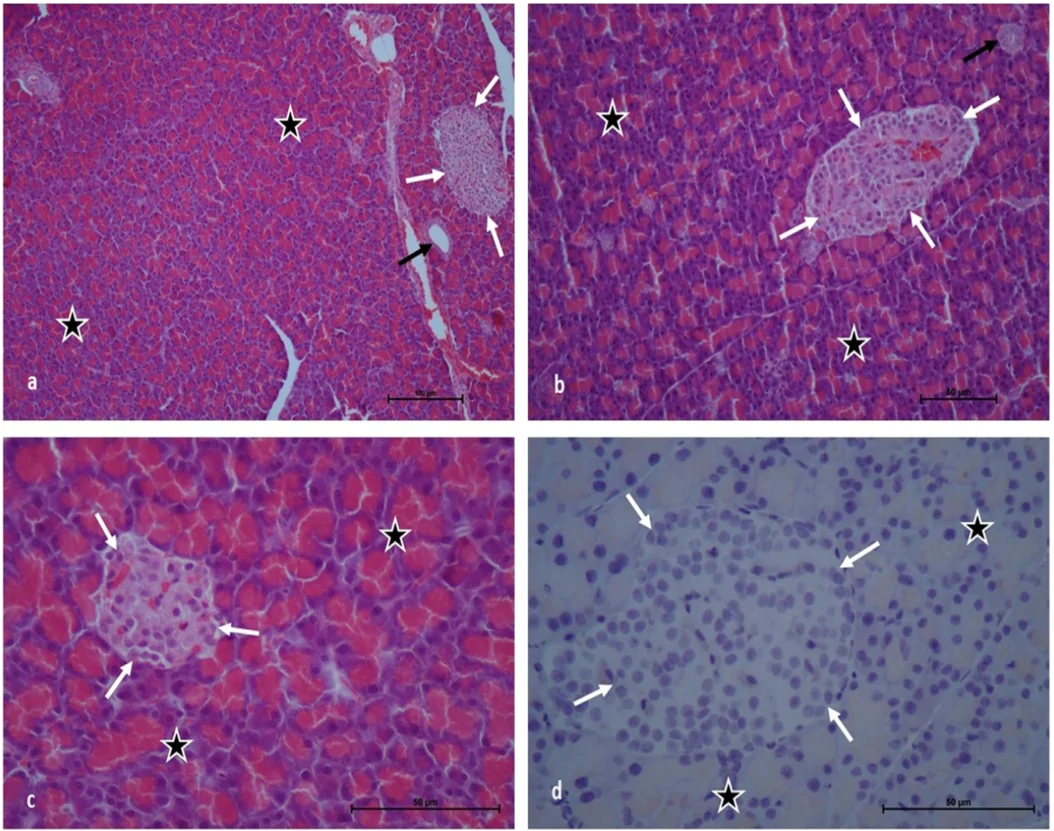
ω−3 group histopathological images. (a) Omega‐3 group: Endocrine pancreas islet (white arrows) and exocrine pancreas (aster), pancreatic duct (black arrow) with normal histological appearance. H&E, X10. (b) Omega‐3 group: Endocrine pancreas (white arrows) and exocrine pancreas (aster) and panreatic duct (black arrow) in normal histological appearance. H&E, X20. (c) Omega‐3 group: Endocrine pancreas islet (arrows) and exocrine pancreas acini (aster) in normal histological appearance. H&E, X40. (d) Omega‐3 group: Negative caspase‐3 immunoreactivity in endocrine pancreatic cells (arrows) and exocrine pancreatic acinar cells (aster) and pancreatic duct epithelial cells (arrow heads). IHC, X40.

In both the Control group (Figure 1d) and the Omega‐3 group (Figure 2d), sections stained with an anti‐caspase‐3 antibody showed that caspase‐3 immunoreactivity was generally negative in exocrine and endocrine pancreatic cells. However, some areas exhibited low levels of weak positive immunoreactivity. Significant histopathological changes were seen in both the exocrine and endocrine pancreas in haematoxylin and eosin (H&E)‐stained pancreatic sections from the DM group. In the exocrine pancreas, the histological structure of the acini and the islets of the endocrine pancreas was damaged. Marked intrascytoplasmic vacuolization, widespread acinar cell damage, and partial or complete acinar degeneration were observed in the parenchymal areas of the exocrine pancreas. The pancreatic endocrine cells of the islets showed heterochromatic‐pyknotic nuclei, dense eosinophilic cytoplasm in some cells, and hydropic vacuolar changes in others (Figure 3a–c). The DM group also showed high intensity and prevalence of positive staining in both exocrine pancreatic acinar cells and endocrine pancreatic cells in sections treated with anti‐caspase‐3 (Figure 3d). Histopathological damage scores for both the endocrine and exocrine pancreas, as well as anti‐caspase‐3 immunoreactivity H scores, were significantly higher in pancreatic sections from the DM group compared to those from the control and Omega‐3 groups (Table 5).

**Figure 3:**
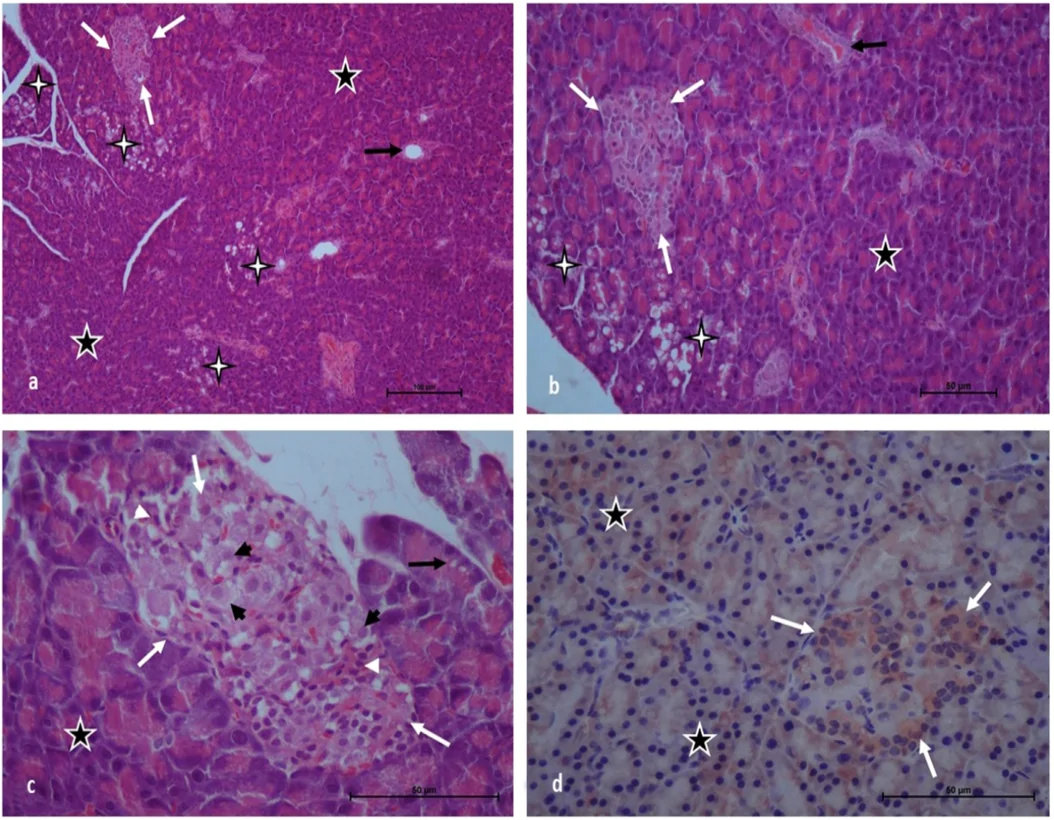
Diabetes Mellitus group histopathological images. (a) DM group: Endocrine pancreas islet (white arrows) and exocrine pancreas (five‐point aster), pancreatic duct (black arrow), vacuolisation in acinar cells and acinar damage/degeneration (four‐point aster). H&E, X10. (b) DM group: Endocrine pancreas islet (white arrows) and exocrine pancreas (five‐point aster), pancreatic duct (black arrow), vacuolisation in acinar cells and acinar damage/degeneration (four‐point aster). H&E, X20. (c) DM group: Endocrine pancreas islet (white arrows) and exocrine pancreas (aster), intracellular vacuoles in asiner cells (black arrow). Dense eosinophilic cytoplasm and heterochromatic pyknotic nuclei (white arrow) and hydropic‐vacuolar cytoplasm (black arrowhead) in endocrine pancreatic cells. H&E, X40. (d) DM: Distinc positive caspase‐3 immunoreactivity in endocrine pancreatic cells (arrows) and exocrine pancreatic acinar cells (aster). IHC, X40.

**Table 5:** Caspase‐3 immunoreactivity H score parameters.

	Caspase‐3 Immunoreactivity H score of endocrine pancreatic cells	Caspase‐3 Immunoreactivity H score of exocrine pancreatic tissus
Control	1^a^ ± 0	1^a^ ± 4
ω−3	1^a^ ± 0	1^a^ ± 3
DM	110^b^ ± 50	65^b^ ± 20
DM + ω−3	15^c^ ± 17.5	10^c^ ± 10
*p*‐value	< 0.001	< 0.001

*Note:* Data are expressed as mean ± standard deviation. There is a statistically significant difference between groups marked with different superscript letters.

H&E‐stained pancreatic tissue sections from the DM + omega‐3 group showed significant improvements in histopathological damage in both the endocrine and exocrine areas of the pancreas (Figure 4a–c). In this group, the damage scores for the endocrine and exocrine pancreatic tissues, as well as the H‐scores for anti‐caspase‐3 immunoreactivity, were significantly lower than those in the DM+omega‐3 group but higher than in the control and omega‐3 groups (Figure 4d, Table 6).

**Figure 4:**
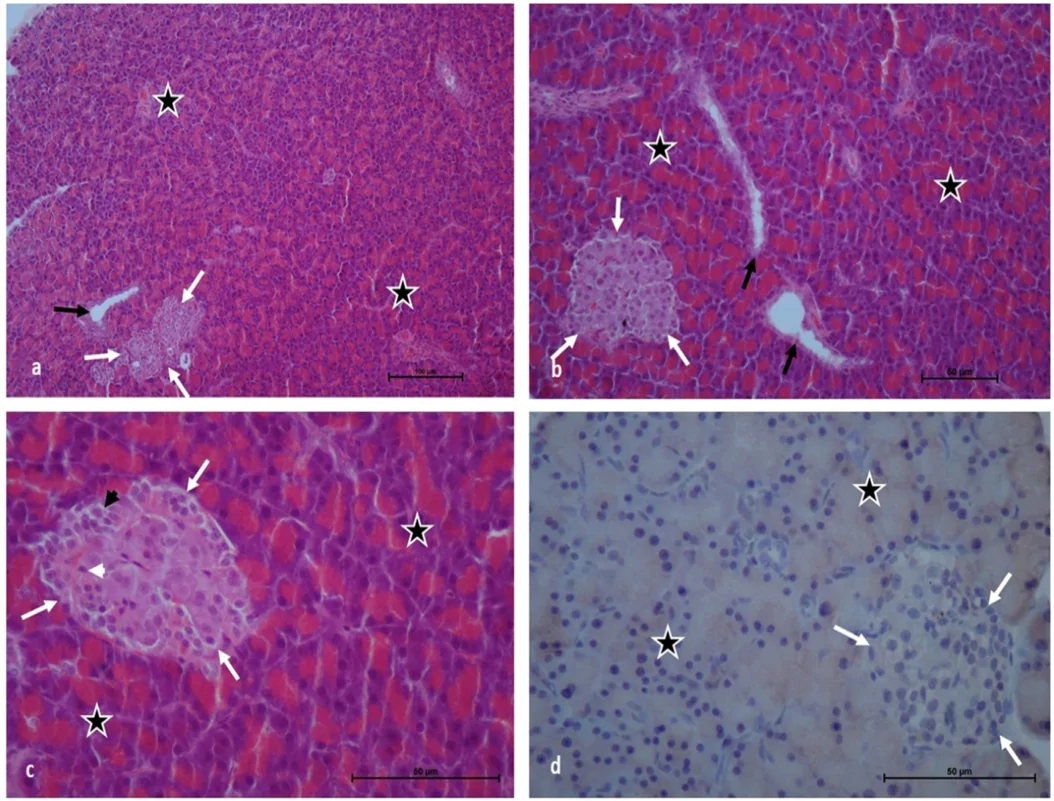
ω−3 + Diabetes Mellitus group histopathological images. (a) DM+Omega‐3 group: Normal histological appearance of the endocrine (white arrows) and exocrine (aster) pancreas, pancreatic duct (black arrow). H&E, X10. (b) DM+Omega‐3 group: Endocrine pancreas (arrows) and exocrine pancreas (aster) and panreatic duct (arrow heads) in normal histological appearance. H&E, X20. (c) DM+Omega‐3 group: Normal histological appearance of exocrine pancreas (aster), Dense eosinophilic cytoplasm and heterochromatic pyknotic nuclei (white arrow) and hydropic‐vacuolar cytoplasm (black arrowhead) in endocrine pancreatic cells. H&E, X40. (d) DM+Omega‐3 group: Weak positive caspase‐3 immunoreactivity in endocrine pancreatic cells (arrows) and exocrine pancreatic acinar cells (aster). IHC, X40.

**Table 6:** Total damage score parameters.

	Histopathological damage in endocrine pancreatic cells	Histopathological damage in exocrine pancreatic tissue	Total histopathological damage score
Control	0^a^ ± 0	0^a^ ± 0	0^a^ ± 0
ω−3	0^a^ ± 0	0^a^ ± 0	0^a^ ± 0
DM	2^b^ ± 1	2^b^ ± 1	4^b^ ± 0.75
DM + ω−3	0.5^c^ ± 1	0^c^ ± 1	1^c^ ± 0.75
*p*‐value	< 0.001	< 0.001	< 0.001

*Note:* Data are expressed as mean ± standard deviation. There is a statistically significant difference between groups marked with different superscript letters.

## 4 Discussion

Diabetes mellitus, commonly called diabetes, is a long‐term disease caused by either a lack of insulin production by the pancreas or the body's inability to use insulin effectively, leading to high blood sugar levels. DM results in increased oxidative stress due to the overproduction of ROS and decreased antioxidant defenses. Studies have shown a significant reduction in antioxidant enzymes and an increase in lipid peroxidation in both Type 1 and Type 2 DM animal models. Research using STZ‐induced rats has demonstrated a notable decrease in antioxidant enzymes such as SOD, CAT, and GSH activities, along with a significant increase in MDA levels, a marker of lipid peroxidation [25].

Li et al. developed a DM model in rats by administering 35 mg/kg STZ and examined pancreatic tissues. In the diabetes group, they observed increased levels of blood glucose, total cholesterol, triglycerides, LDL, and VLDL, along with decreased SOD, CAT, and GSH levels compared to the control group. They also noted pancreatic histopathological abnormalities in the DM group [26]. Hazer‐Yavuz et al. reported that, in a DM model where they administered 55 mg/kg STZ to rats, GSH levels decreased and MDA levels increased in pancreatic tissue compared to the control group [27]. Yen et al. created an STZ‐induced diabetes model in rats and found that the diabetes group had higher glucose, LDL, and triglyceride levels, while insulin and HDL levels were lower than in the control group. Histopathological abnormalities in pancreatic tissue were also observed [28]. Ahmed et al. administered 40 mg/kg STZ to develop a DM model in rats. Koroglu et al. reported increased glucose, IL‐6, and TNF‐α levels, along with decreased SOD, CAT, and GSH levels, and deterioration of pancreatic tissue histology in the diabetes model compared to controls [29]. Koroglu et al. used 65 mg/kg STZ to induce diabetes in rats. In the group with diabetes, they reported a decrease in GSH and TAS levels, an increase in TOS, OSI, TNF‐α, and IL‐6 levels, as well as deterioration in pancreatic tissue and an increase in histopathological error score compared to the control group [30]. They reported that all STZ administrations induced diabetes and caused deterioration of pancreatic tissue, and a decrease in insulin secretion, especially with the deterioration of the Langerhans islets in the pancreas. They explained that, with decreased insulin secretion, glucose cannot be taken up by cells, disrupting normal cellular processes and triggering oxidative stress. They argued that ongoing oxidative stress also triggers inflammation, leading to pancreatic tissue damage. Our results are similar to those of the researchers. Oxidant capacity increased, and antioxidant capacity decreased, resulting in oxidative stress and inflammation. Additionally, histopathological abnormalities were observed in the pancreas. Our biochemical and histopathological findings are consistent, confirming that STZ induced diabetes and caused pancreatic damage.

ω−3 is a long‐chain polyunsaturated fatty acid that supports various bodily functions and is a key component of the cell membrane. The main benefits of ω3 include strengthening immunity, improving cardiovascular health by increasing HDL levels, reducing inflammation, promoting eye and brain health, lowering triglyceride and blood pressure levels, alleviating symptoms of rheumatoid arthritis, and enhancing mental health by reducing depression effects. Studies on experimental animals have demonstrated protective effects against reproductive toxicity, neurotoxicity, and toxicity in the liver, kidney, and pancreas [31, 32, 33]. Hamden et al. created an alloxan‐induced diabetes model in male rats and administered ω−3 fatty acids as a protective agent. They observed that, compared to the control group, blood sugar, LDL, triglycerides, and total cholesterol increased in the diabetes group, and β‐cell atrophy appeared in pancreatic tissue. They reported that ω3 supplementation reduced these adverse effects in both serum and pancreatic tissue [34]. Zou et al. treated rats with ω3 as a preventive measure for 30 days in a 150 mg/kg STZ‐induced diabetes model. At the end of the study, they found increased MDA levels, decreased SOD and CAT enzyme levels, and histopathological abnormalities in the STZ‐treated group, which were mitigated by ω3 administration [35]. Safhi et al. administered 300 mg/kg ω3 as a preventive for 28 days in a 60 mg/kg STZ‐induced diabetes model. They noted significant increases in glucose, insulin, triglycerides, and LDL levels, along with decreased HDL, in the diabetes group compared to all other groups. They also observed histopathological abnormalities and reported that ω3 administration eliminated these negative effects [36]. Titisari et al. induced diabetes by giving 45 mg/kg STZ and administered fish oil at doses of 1 and 3 g/kg for 3 weeks as a protective measure. They reported elevated insulin levels and deterioration of pancreatic tissue in the diabetes group, but these effects were reversed with dose‐dependent fish oil treatment [37]. Ayuob et al. used ω−3 for 4 weeks as a shield against energy drinks. At the end of this period, they examined rat pancreatic tissues and found that, compared to other groups, the ω3 group showed lower blood glucose levels, reduced caspase 3 immunoreactivity, and histopathological features similar to the control group [38]. Our findings align with these studies. ω3 prevents the overproduction of reactive oxygen species like O_2_‐, OH‐, and H_2_O_2_, thereby inhibiting the increase in oxidative stress. It not only suppresses oxidative capacity but also boosts antioxidant defenses, reducing oxidative stress. Excessive oxidative capacity can damage cells, triggering cytokine production. TNF‐α and IL‐6 are among the key pro‐inflammatory cytokines, and their increase promotes lipid peroxidation. Additionally, ω3 has been shown to suppress inflammation. ω−3 enhances antioxidant capacity, suppresses oxidant activity, and prevents oxidative stress. While these findings contribute to the scientific literature through biochemical, histological, immunohistochemical, and inflammatory data, further research on the dosage, frequency of use, and method of administering ω−3 and STZ will help fill existing knowledge gaps.

## 5 Conclusion

The rapid global spread of diabetes is an undeniable reality. The burden that diabetes and its complications place on individuals and healthcare systems continues to grow. Our research suggests that ω−3 therapy may help lessen negative effects on diabetic pancreatic tissue and provide a protective role. However, larger and long‐term studies are needed to verify these findings and thoroughly assess ω−3's effectiveness.

## Author Contributions

**Zeynep Erdemli:** visualization, methodology. **Mehmet Gul:** methodology. **Nurcan Gokturk:** investigation, formal analysis. **Fatma Ozhilal:** formal analysis. **Edanur Ozsoy:** investigation, formal analysis. **Harika Gozukara Bag:** data curation. **Mehmet Erman Erdemli:** writing – review and editing, writing – original draft.

## Funding

The authors have nothing to report.

## Ethics Statement

The study received approval from the Experimental Animals Ethics Committee at Inonu University, Faculty of Medicine (Protocol No: 2022/12‐2).

## Conflicts of Interest

The authors declare no conflicts of interest.

## Data Availability

The data sets used and/or analyzed during the current study areavailable from the corresponding author on reasonable request.
